# Intra-Facility Linkage of HIV-Positive Mothers and HIV-Exposed Babies into HIV Chronic Care: Rural and Urban Experience in a Resource Limited Setting

**DOI:** 10.1371/journal.pone.0115171

**Published:** 2014-12-29

**Authors:** Christine Mugasha, Joanita Kigozi, Agnes Kiragga, Alex Muganzi, Nelson Sewankambo, Alex Coutinho, Damalie Nakanjako

**Affiliations:** 1 Infectious Disease Institute, Kampala, Uganda; 2 Makerere University College of Health Sciences, Kampala, Uganda; London School of Hygiene and Tropical Medicine, United Kingdom

## Abstract

**Introduction:**

Linkage of HIV-infected pregnant women to HIV care remains critical for improvement of maternal and child outcomes through prevention of maternal-to-child transmission of HIV (PMTCT) and subsequent chronic HIV care. This study determined proportions and factors associated with intra-facility linkage to HIV care and Early Infant Diagnosis care (EID) to inform strategic scale up of PMTCT programs.

**Methods:**

A cross-sectional review of records was done at 2 urban and 3 rural public health care facilities supported by the Infectious Diseases Institute (IDI). HIV-infected pregnant mothers, identified through routine antenatal care (ANC) and HIV-exposed babies were evaluated for enrollment in HIV clinics by 6 weeks post-delivery.

**Results:**

Overall, 1,025 HIV-infected pregnant mothers were identified during ANC between January and June, 2012; 267/1,025 (26%) in rural and 743/1,025 (74%) in urban facilities. Of these 375/1,025 (37%) were linked to HIV clinics [67/267(25%) rural and 308/758(41%) urban]. Of 636 HIV-exposed babies, 193 (30%) were linked to EID. Linkage of mother-baby pairs to HIV chronic care and EID was 16% (101/636); 8/179 (4.5%)] in rural and 93/457(20.3%) in urban health facilities. Within rural facilities, ANC registration <28 weeks-of-gestation was associated with mothers' linkage to HIV chronic care [AoR, 2.0 95% CI, 1.1–3.7, p = 0.019] and mothers' multi-parity was associated with baby's linkage to EID; AoR 4.4 (1.3–15.1), p = 0.023. Stigma, long distance to health facilities and vertical PMTCT services affected linkage in rural facilities, while peer mothers, infant feeding services, long patient queues and limited privacy hindered linkage to HIV care in urban settings.

**Conclusion:**

Post-natal linkage of HIV-infected mothers to chronic HIV care and HIV-exposed babies to EID programs was low. Barriers to linkage to HIV care vary in urban and rural settings. We recommend targeted interventions to rapidly improve linkage to antiretroviral therapy for elimination of MTCT.

## Introduction

Globally, 34 million people are living with HIV/AIDS, with 69% living in sub-Saharan Africa (SSA) by the end of 2010 [1]. Women living with HIV/AIDS contribute 56% of the HIV/AIDS burden in SSA. Maternal to child transmission of HIV (MTCT) contributes 390,000 new HIV infections in children out of 2.7 million new HIV infections [1]. In Uganda, 1.4 million people were estimated to be living with HIV, of whom 670,000 (48%) were women and 190,000 (14%) were children [2]. MTCT rates are still high and early Infant diagnosis (EID) coverage is still as low as 35% in 21 countries including Uganda [3]. Achieving the global goal of reducing the number of new pediatric HIV infections by 2015 requires great efforts to link pregnant women and children to HIV care and treatment [1].

Uganda has made remarkable milestones in her response to the HIV epidemic among women, as evidenced by established HIV counseling, testing and PMTCT services at 81% of all the national health facilities [4]. Although 72% of pregnant women were tested for HIV and received their results as part of antenatal care (ANC), in 2011, only half of HIV-infected pregnant mothers received efficacious antiretroviral therapy (ART) regimens for prevention of maternal to child transmission (PMTCT) and 20% of HV-exposed infants received antiretroviral drugs for PMTCT [1], [3]–[8]. In addition, EID coverage is still 33% among HIV-exposed infants [1]. Thus, elimination of MTCT requires critical evaluation and strengthening of weak links in the PMTCT cascade from screening during ANC to long-term ART for mothers post-delivery and EID for HIV-exposed babies.

Uganda adopted use of single dose nevirapine for PMTCT in 2002, transitioned to option A (initiating zidovudine as early as 14 weeks of pregnancy, single dose nevirapine during labour and combivir for a week postpartum for mother while infant receives nevirapine syrup during breastfeeding or up to 1 year) in 2010, and adopted option B plus for PMTCT (ART initiation for HIV-infected pregnant and lactating mothers for life) in 2012. A national cohort review of Uganda's PMTCT program in 2010 revealed that only 8% of HIV-infected pregnant mothers (and their exposed infants), completed the entire PMTCT cascade with mothers active in HIV care post-pregnancy and DNA polymerase chain reaction [PCR] done for exposed babies [9].

Effective prevention of new paediatric infections requires adherence of mothers and infants to the full spectrum of PMTCT interventions from pregnancy till cessation of breast feeding. Therefore, adherence to ART and retention of mothers and mother-baby pairs post-delivery remains essential for prevention of HIV transmission to infants as well as improvement of mothers' HIV treatment outcomes. A majority of surveys on uptake of PMTCT have described individual and cultural factors affecting effective PMTCT in resource-limited settings; highlighting non-disclosure, discrimination, stigma, fear of being tested, limited access to health facilities, lack of male partner support, and difficulties with infant feeding and health worker attitudes [10]–[14]. Despite efforts to increase access to comprehensive HIV/AIDS care services including PMTCT and EID within primary urban and rural health care facilities [15], there is limited data on barriers to linkage of HIV-infected mothers and HIV-exposed babies to available HIV prevention, care and treatment services within individual health care facilities. We evaluated intra-facility linkage of HIV-infected mothers and HIV-exposed infants to HIV chronic care and EID in rural and urban health care facilities in Uganda. Our findings inform the development of strategic interventions to increase uptake and adherence to PMTCT interventions, in line with the national goal to eliminate new pediatric HIV infections.

## Methods

### Study Design

In a descriptive cross-sectional review of health care facility records at selected urban and rural IDI-supported facilities, clients enrolled into the PMTCT programs between January and June 2012, were evaluated for enrollment into chronic HIV care clinics for mothers and early infant diagnosis (EID) by six weeks post-delivery.

### Study setting and participants

This study was undertaken within the Infectious Diseases Institute (IDI) outreach project for health systems' strengthening of HIV/AIDS care programs at lower-level health care facilities in Kampala city (urban) and Kibaale district (rural mid-western Uganda). The IDI-supported facilities offer free health services including chronic HIV care and antiretroviral therapy (ART), TB/HIV co-infection services, ANC, labor and delivery services, ART for PMTCT and EID for HIV-exposed babies, provided within the national health care structure. All HIV-positive pregnant mothers are registered daily in ANC registers at their first ANC visit. Registered mothers are referred to return for enrolment at the next HIV clinic day (thrice a week to once a month depending on the total HIV patient load at the facility). In urban facilities patients received referral letters and guidance by a mother who had gone through the system before (peer mother). In rural facilities, mothers received referral letter and directions to the HIV clinic. Upon honoring the referral to HIV clinic, pregnant mothers are registered in the HIV clinic pre-ART registers at the first visit. On the same day, pre-ART mothers are initiated on cotrimoxazole prophylaxis and ART (if CD4 counts are ≤350 cells/ul) and added to the ART register. All HIV-infected mothers are expected to attend at least 4 ANC visits, as recommended by the MCH integrated national guidelines on ART and PMTCT, and independent HIV clinic visits. At delivery, mothers receive a post natal care (PNC)-appointment and a referral of the baby for EID, for presentation at six weeks. Mothers receive referral letters and directions to the respective PNC and EID clinics that run independently. After six weeks post-delivery, mothers return for a PNC visit where family planning, routine immunization and growth monitoring are done. On a different day, the mother brings her baby for registration into the EID program, where babies are registered using the mother's name and address.

Among the IDI-supported districts, Kampala, the capital city, was selected to represent the urban setting while Kibaale district was selected randomly out of the 7 rural IDI-supported districts. In Kampala, two HIV/AIDS care facilities (Kawempe HC IV and Komamboga HC III) with the largest numbers of mothers, were conveniently selected out of eight IDI-supported facilities and in mid-western Uganda, three largest rural facilities (Kagadi Hospital, Kibaale HC IV and Nyamarwa HC III), were conveniently selected out of 22 rural IDI-supported HIV/AIDS care facilities.

### Procedures

Pre-tested, pre-coded data extraction tools were used to collect and track demographic and clinical data for enrolled HIV-infected mothers and HIV-exposed infants recorded in the ANC, pre-ART and EID registers. Using mothers' names and at least 2 other demographic identifiers such as age and address, mothers in the ANC register were tracked for enrolment in the ART register and mother's name was used to track HIV-exposed infants for registration in the EID registers. The variables recorded included mothers' age, parity, access to a phone contact, gestation age at ANC registration, ART status, date of ART initiation, baby's EID registration status and date of registration, as well as mother-baby HIV clinic registration.

### Operational definitions

**Intra-facility linkage of HIV-infected mothers to chronic HIV care**, was defined as the proportion of HIV-positive mothers recorded in the ANC and labor registers (for mothers whose first visit occurred during labor), that appeared in the facility pre-ART register and had a clinic number with at least one clinical visit recorded by six weeks post-delivery.

**Intra-facility linkage of HIV-exposed babies to the EID program** was defined as the proportion of babies born to HIV-infected mothers (as per ANC and labor registers), that were registered in the facility EID register with at least one clinical visit recorded by a clinician, by six weeks post-delivery.

**Intra-facility linkage of mother-baby pairs** was defined as the proportion of mother-baby pairs (identified from the ANC and labor registers), that had the HIV-infected mother registered in the HIV clinic and the baby registered in the EID program by 6 weeks post-delivery.

To assess factors associated with intra-facility linkage to HIV chronic care and EID, a pre-tested self-administered questionnaire, with open-ended questions, was administered to health care providers (midwives, nurses, doctors, clinical officers, laboratory staff, in the ANC, postnatal, labor, immunization, EID clinics) including facility heads as key informants to determine health worker perspectives of promoters and hindrances to linkage of mothers and babies within their respective health care facilities. In addition, ten focus group discussions (FGD) were conducted among health workers to further understand health worker perspectives on determinants and hindrances to intra-facility linkage to chronic HIV care and EID following PMTCT interventions at the respective health care facilities. Each FGD consisted of 4–8 people including a nurse, midwife, doctor, peer mother, laboratory technicians and clinical officers. The FGD guide included pre-determined themes that were developed through monitoring and evaluation meetings by the IDI outreach team in charge of PMTCT program implementation. The themes covered health worker factors, health system factors and patient factors affecting linkage of mothers and babies to HIV care after PMTCT, from the perspectives of health workers. All FGDs occurred concurrently at different sites and each FGD had a leader and a note taker. This work was done in compliance with the Helsinki Declaration (http://www.wma.net/en/30publications/10policies/b3/index.html), and all health workers (key informants and FGD members) provided verbal informed consent. Ethical approval was provided by the Makerere University College of Health Sciences, School of Medicine review board.

### Statistical Analysis

Quantitative data was entered using Microsoft Excel and exported to STATA version 11.0 for analysis. Demographic and clinical data were summarized using frequencies and medians. Proportions of intra-facility linkage of HIV-infected mothers to HIV care, HIV-exposed children to EID and mother-baby pairs were presented as per case definitions. Student T test was used to compare continuous variables and Pearson's Chi square test was used to compare categorical variables among individuals linked and those that were not linked to HIV care and EID programs by six weeks post-delivery. All variables were entered in a stepwise backward multivariate logistic regression model to determine predictors of getting linked to HIV care and EID within each of the studied health care facilities. P-values <0.05 were considered statistically significant. Qualitative data from FGDs and key informant questionnaires was analyzed using the principles of thematic analysis [16], [17]. Data content was analysed manually according to the predetermined themes; summarised in tables and quotes were reported verbatim. The analysis themes included motivators and hindrances to intra-facility linkage to HIV care such as health worker, health systems and social support factors, as perceived by the health workers. Content analysis included access to services, health worker competence, as well as patients' perceptions and social structure. All data was stored securely with access limited to the study team.

## Results

### Study population characteristics

**Figure 1:**
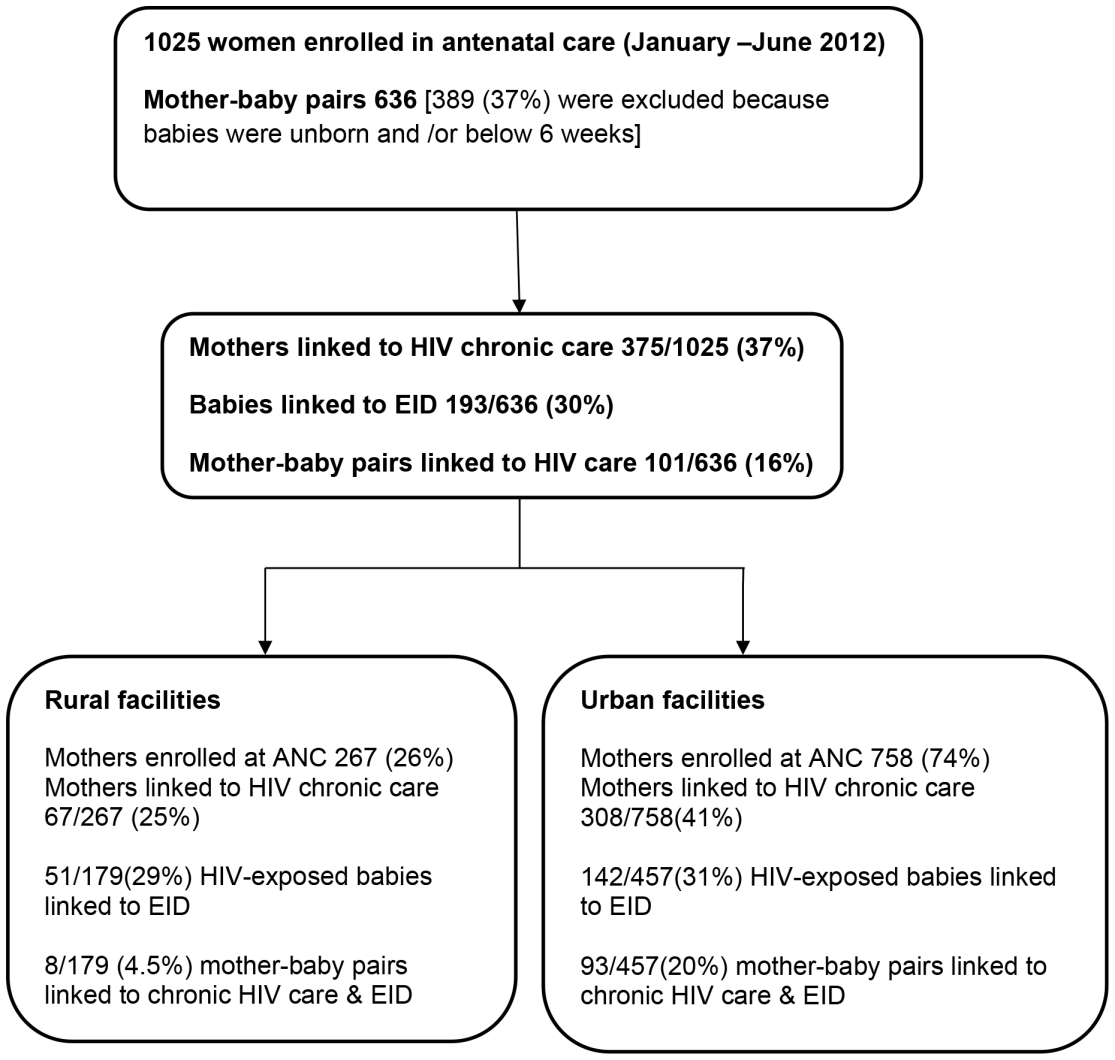
HIV-infected mothers and exposed infants followed up for six weeks post-delivery within the national PMTCT program. **Mothers ‘linked' to HIV/AIDS care** were HIV-positive mothers recorded in the antenatal register, that appeared in the facility pre-ART register and had a clinic number with at least one clinical visit recorded by a clinician six weeks post-delivery. **Babies ‘linked' to Early Infant Diagnosis (EID)** were babies born to HIV-infected mothers (as per antenatal and labor registers), that were registered in the facility EID register with at least one clinical visit recorded by a clinician by six weeks post-delivery. **Mother-baby pairs ‘linked'** were mother-baby pairs identified from the antenatal and labor ward registers, that had the HIV-infected mother registered in the HIV clinic and the baby registered in the EID program by 6 weeks post-delivery. Babies ‘not eligible' were unborn and/or below 6 weeks of age at the time of the study were excluded.

Overall, 1,025 HIV-positive pregnant women were enrolled at the five selected antenatal clinics from January to June 2012. Of these, 267 (26%) were identified in rural health facilities. Ineligible mother-baby pairs [389 (37%)] were excluded because babies were unborn and/or below 6 weeks (Fig. 1). Mothers in rural health facilities were older [median age 26, interquartile range (IQR), 22–31 years in rural versus median 25 (IQR 22–29) years in urban settings], had more children [median parity, 3 (IQR 2–4) in rural versus 2 (IQR 2–4) in urban facilities], and registered earlier for antenatal care (54% in rural versus 27% in urban facilities registered in ANC before 24 weeks of gestation), than mothers in urban settings (Table 1).

**Table 1:** Demographic and clinical characteristics of HIV-infected pregnant mothers evaluated for linkage into HIV chronic care within rural and urban health care facilities.

Variables	Rural health facilities N = 267 (26%)	Urban health facilities N = 758 (74%)	P-value*
Age, Median (IQR) years	26 (22–31)	25 (22–29)	0.001
Phone access, n (%)	29 (11)	229 (30)	<0.001
Parity, Median (IQR) pregnancies	3 (2–4)	2 (2–4)	<0.001
ANC registration^#^			
<24 weeks of gestation, n (%)	133 (54)	144 (27)	<0.001
ARV use, n (%)	225 (84)	199 (26)	<0.001

*Student's T test was used to compare continuous variables and Pearson's Chi-square test was used to compare the proportions of categorical variables. # 246 mothers in rural health facilities and 533 mothers in urban were considered under weeks of gestation.

### Intra-facility linkage to HIV chronic care

#### HIV infected pregnant women

Overall, 375/1,025 (37%) of HIV-infected pregnant mothers identified in ANC, were linked to HIV chronic care (Fig. 1). Of these, 67/267(25%) were from rural facilities with a median time of 0 (IQR 0–8) days from ANC registration to enrolment into HIV chronic care, and 308/758(41%) were from urban health care facilities, with a median time of 5 (IQR 1–42) days from ANC registration to enrolment into HIV chronic care.

#### HIV-exposed babies

Overall, 193/636 (30%) of HIV-exposed babies were linked to EID programs. Of these, 51/179 (29%) were from rural health facilities and 142/457 (31%) were from urban settings (Fig. 1).

#### Mother-baby pairs

Overall, 101/636(16%) mother-baby pairs were linked to HIV care and EID. Of these, 8/179 (5%) were from rural facilities and 93/457(20%) were from urban facilities (Fig. 1).

### Factors associated with linkage to chronic HIV/AIDS care and EID after PMTCT

#### HIV-infected mothers

Within rural health care facilities, HIV-infected mothers that registered for ANC before 28 weeks of gestation were more likely to be linked to HIV chronic care by six weeks post-delivery [Adjusted odds ratio (AoR), 2.0 95% confidence interval (CI), 1.1–3.7, p = 0.019]. In addition, mothers with 2 or more previous pregnancies were more likely to be linked to HIV care than first-time mothers (prime gravida), although the difference was not statistically significant (p = 0.064). Mothers' age, access to a phone and parity were not associated with linkage to chronic care among the women in the rural setting. In the urban setting, there were no significant differences among mothers that were linked or not linked to chronic HIV care (Table 2).

**Table 2:** Factors associated with linkage of mothers to HIV chronic care after PMTCT within rural and urban health care facilities.

Characteristics	Category	Mothers Linked	Mothers not Linked	Adjusted odds ratio, (95% CI)	P-value
		RURAL	N = 267		
**Mothers' linkage to care**		68 (25%)	199 (75%)		
**Age**	≤25 years	35 (51)	87 (44)	1	
	>25 years	33 (49)	112 (56)	0.7 (0.4–1.3)	0.269
**Phone Access**	No phone contact	61 (90)	177(89)	1	
	Has phone contact	7 (10)	22 (11)	0.9 (0.4–2.3)	00.862
**Parity**	Multi para	53 (78)	174 (87)	1	
	Prime para	15 (22)	25 (13)	2.0 (1.0–4.1)	00.064
**Gestation ¥** ¥	≤24 weeks	45 (69)	97 (49)	1	
	>24 weeks	20 (31)	87 (44)	2.0 (1.1–3.7)	00.019
**ART use**	Using ARVs	54 (79)	170 (85)	1	
	None	14 (21)	29 (15)	0.8(0.6–1.1)	00.246
		**URBAN**	**N = 758**		
**Mothers' linkage to care**		**308 (41%)**	**450(59%)**		
**Age** ♯	≤25 years	165 (55)	245 (56)	1	
	>25 years	136 (45)	196 (44)	1.0 (0.8–1.4)	00.843
**Phone Access**	No phone contact	210 (68)	319 (71)	1	
	Has phone contact	98 (32)	131 (29)	1.1 (0.8–1.6	00.425
**Parity** †	Multi para	240 (79)	353 (80)	1	
	Prime para	63 (21)	89 (20)	1.0 (0.7–1.5)	00.827
**Gestation** *	>24 weeks	135 (44)	190 (49)	1	
	≤24 weeks	76 (36)	131 (41)	0.8 (0.6–1.2)	00.268
**ART use**	Using ART	86 (28)	113(25)	1	
	None	222 (72)	337(75)	2 (0.8–1.6)	00.388

ART, anti-retroviral therapy,

¥ 18 rural mothers missed parity data,

♯ 16 urban mothers missed age data,

† 13 urban mothers missed parity data,

*226 urban mothers had no data on gestation age at ANC registration.

#### HIV-exposed babies

Within rural facilities, multi-parous mothers (presenting with third pregnancy) were more likely to have their babies linked to the EID program by six weeks post-delivery; AoR 4.4 (1.3–15.1), p = 0.023. In the urban setting, there was no difference between mothers whose babies were linked and mothers whose babies were not linked to EID programs (Table 3).

**Table 3:** Factors associated with linkage of babies to the early infant diagnosis (EID) program after PMTCT within rural and urban health care facilities.

Characteristic	Category	Babies Linked	Not Linked	AoR (95% CI)	P value
**RURAL N = 179**					
**Linkage to EID**		**51 (29%)**	**128 (71%)**		
**Age n (%)**	≤25 years	17 (33)	60 (47)	1	
	>25 years	34 (67)	68 (53)	1.8(0.9–3.5)	0.101
**Phone Access n (%)**	No phone contact	43 (84)	111 (87)	1	
	Has phone contact	8 (16)	17 (13)	1.2 (0.5–3.0)	0.676
**Parity †n (%)**	Multi para	47(94)	100 (78)	1	
	Prime para	3 (6)	28 (22)	4.4 (1.3–15.1)	0.023
**Gestation¥ n (%)**	≤24 weeks	31 (63)	74 (58)	1	
	>24 weeks	18 (37)	54 (42)	1.3 (0.6–2.5)	0.509
**ARV use n (%)**	Using ARVs	45 (88)	115 (90)	1	
	None	6 (12)	13 (10)	0.8 (0.3–2.4)	0.753
		**URBAN N = 457**			
**Linkage to EID**		**142 (31%)**	**315 (69%)**		
**Age** ♯ **n (%)**	>25 years	69 (49)	160 (51)	1	
	>25 years	72 (51)	151 (49)	1.1 (0.7–1.6)	0.621
**Phone Access**	No phone contact	99 (70)	200 (67)	1	
	Has phone contact	43 (30)	115 (73)	0.8 (0.5–1.2)	0.196
**Parity** †	Multi para	120 (85)	250 (80)	1	
	Prime para	22 (15)	63 (20)	1.4 (0.8–2.3)	0.241
**Gestation** *	≤24 weeks	67 (77)	240 (77)	1	
	>24 weeks	20 (23)	73 (23)	1.0 (0.6–1.8)	0.948
**ARV use**	Using ART	36 (25)	106 (34)	1	
	None	106 (75)	209 (66)	0.7 (0.5–1.0)	0.077

**≈**1 missing record on mothers' parity,

¥ 2 records missing mothers' gestation age at ANC registration, among rural health care facilities.

♯ 4 records missing age,

† 2 records missing parity,

* 57 records missing gestation age at ANC registration, AoR - Adjusted odds ratio.

### Health worker perspectives on factors influencing linkage of mothers and babies to chronic HIV/AIDS care and EID programs after PMTCT

In general, health workers felt that high health worker competence in counseling skills influenced mothers' uptake of HIV/AIDS care services during the post-natal period and beyond (Table 4).

**Table 4:** Factors influencing intra-facility linkage of HIV-infected mothers and HIV-exposed babies to chronic HIV/AIDS care and early infant diagnosis programs in central and mid-western Uganda.

Sphere of Influence	Motivators for intra-facility linkage	Hindrances to intra-facility Linkage
**Health worker factors**	• Health workers' competence in counseling clients to enroll into the PMTCT and chronic care programs	• Limited time available to provide post-test counseling for enrollment into HIV chronic care programs in addition to PMTCT care.
**Perceived Client factors**	• Availability of HIV counseling and testing, PMTCT, EID and HIV chronic care services at the facilities	• Mothers' fear to disclose HIV positive status to partners
	• Benefits of PMTCT program, for example early HIV diagnosis for infants and HIV negative infants	• Mothers' stigma
		• Low rates of health worker-attended deliveries
**Health system factors**	• Availability of private rooms to counsel patients to enroll into chronic HIV care	• Lack of protocols that address linkage between HIV care points at the facilities
	• Availability of free HIV care services	• Long waiting times at ART clinics and EID care points
**Access to services**	• Availability of immunization and EID services on the same day to encourage enrollment of babies	• Long distances to the health units with limited public transportation
	• Provision of infant feeding and nutrition services to motivate mothers' adherence to EID care	• MCH, HIV care and EID services provided in different areas of the health facility
	• Point of care CD4 testing available at the urban sites motivated mothers to enroll into chronic care	• MCH, HIV care and EID services offered on different days of the week
**Social support structure**	• Availability of peer mothers at urban sites to escort clients, provide peer counseling and support was a motivator for linkage	• Low male partner involvement and support

PMTCT- prevention of maternal-to-child transmission of HIV, EID-early infant diagnosis of HIV, MCH- maternal child health.

“Many mothers don't come back after the first visit when they are tested and started on ART. This is because they may still be confused and have not really understood why they need to start and stay on the ART. We therefore need some training in counseling these pregnant women for the test and treat method to work-”, said one health care provider.

Availability of human resource and private rooms for the counseling sessions was noted as a motivator for health workers to provide mothers with the required counseling support and information to improve linkage and utilization of chronic HIV/AIDS care and EID services.

*“Having someone dedicated to provide counseling and start mothers on ART in a separate room within our busy antenatal clinics has been important because mothers still feel stigmatized if other people know their HIV status” said one nurse/counselor.*

In addition, health workers felt that integration of PMTCT and chronic HIV/AIDS care programs within routine maternal child health (MCH) services would improve uptake by HIV-infected mothers and their HIV-exposed babies.

*“Many pregnant women are sent to the HIV clinic building which is well known by the community to provide care for only HIV patients. However many women fear to go to the clinic since they have not disclosed to their partner at home yet they can be seen at the clinic by a neighbor”,*

Health workers felt that HIV-infected mothers would benefit from counseling support and mentoring from peer mothers. ‘*Mothers that have gone through the system would help to guide and encourage our new mothers'* said one nurse/counselor.

On the other hand, health workers felt that the overwhelming numbers of mothers in ANC limits the time health workers use to deliver post-test counseling to mothers that receive HIV-positive result, highlighting that this might hinder mothers' uptake of chronic HIV/AIDS care. *“Although we may see about 8 to 10 new HIV-positive pregnant women a day, we also care for about 200 pregnant women in the antenatal clinic coming in for the first visit or follow up visits. We sometimes are not able to sit for more than 30 min with a pregnant woman who finds it hard to accept her HIV-positive test,”* said one health care provider.

Health workers mentioned that mothers' stigma and fear to disclose HIV infection status to sexual partner(s) and family members, hindered mothers' uptake of chronic HIV care and EID after PMTCT. *“Some mothers find it difficult to take home the ART in case their partners find the medicines and find out they have HIV”* said one health care provider.

Health workers highlighted the lack of unique patient identifiers and protocols to track patients through the various HIV/AIDS care services, including pre-ART prophylaxis of opportunistic infections, counseling, PMTCT, EID, ART, and expert patient programs. “*Because we send HIV-infected women to the ART clinic for chronic care, it is very difficult to confirm their enrollment since many of the mothers may register with other names, or their names may be spelt differently”*, said one health care provider.

Similarly, limited public transport and long distances for mothers to access health care facilities including HIV/AIDS care and other medical consultations was highlighted as a hindrance for mothers to access HIV/AIDS care services (Table 4). One health worker said, *“Many pregnant mothers find it difficult to come for all the required visits and often come once or twice for antenatal care especially during the rainy season, as many of them have to walk for over 5 km to the health facility”*.

Table 4 shows the content analysis of factors influencing uptake of chronic HIV/AIDS care. Mothers' stigma of HIV sero-status, long distance to health facilities and need to integrate PMTCT services with routine MCH services, were highlighted as important. In addition, the role of peer mothers, male involvement, infant feeding services, long patient queues and limited privacy were highlighted as important factors, particularly in the urban settings (Table 4). *“The peer mothers sharing their testimonies with the HIV-infected women attending the clinics may help with mother's accepting to take the ART medicines easily and may also help to reduce loss to follow up especially after the first visit”* said one health worker in Kawempe.

## Discussion

### Linkage of mothers and babies to HIV care and EID after PMTCT

We found that 37% of HIV-infected pregnant women, identified during antenatal care, had been enrolled into HIV chronic care within six weeks post-delivery. In addition, we found that only 30% of HIV-exposed babies were enrolled into the early infant diagnosis (EID) program. These findings were comparable with previous studies in Uganda where 38% of HIV-infected mothers that had deliveries at Mulago national referral hospital, were linked to post-natal PMTCT services by eight weeks post-delivery [14]. Recent reports from the Uganda national PMTCT program showed that 40% of HIV-infected mothers completed the PMTCT cascade and 39% were linked to chronic HIV care at on-site ART clinics [9]. Linkage of HIV-infected mothers to HIV treatment in our study was similar to reports from Malawi where only 45% of HIV-infected pregnant mothers received prophylactic antiretroviral therapy [18]. The 30% enrolment of HIV-exposed babies into EID was comparable with data from Zimbabwe where only 110 (25%) of infants were brought for EID, although 49% of HIV-infected mothers enrolled into the adult HIV clinic [19]. Vertical provision of PMTCT, ART, PNC and EID services at different days and locations could have hindered intra-facility linkage to the available services, and was in-part responsible for discrepancies in linkage of mothers, babies and mother-baby pairs. Mothers needed several visits to the health facility to attend each of the respective clinics independently. The authors therefore recommend integration of routine MCH, PMTCT, EID and chronic HIV care as a strategy to minimize attrition of HIV-infected mothers and their babies. For example, ART for HIV-infected mothers could be provided during ANC visits, and subsequently mothers' ART could be provided at EID clinics until babies get discharged from the EID clinic to a family clinic for ART for mother and/or baby. Therefore, successful implementation of PMTCT Option B plus interventions in developing countries requires tailored innovations to improve proportions of mothers that complete the PMTCT cascade, with initiation of ART, enrolment of HIV-exposed babies into EID program and enrolment into chronic HIV care.

### Factors associated with linkage of mothers and babies to chronic HIV care and EID

More mothers in the urban setting were linked to chronic HIV care (41% in urban and 25% in rural facilities). Only 16% of mother-baby pairs were enrolled into HIV care, with more mother-baby pairs linked to chronic HIV care and EID in urban facilities (20%), than rural facilities (4.5%). More mother-baby pairs were linked to chronic HIV care and EID in urban facilities (20%), than rural facilities (4.5%). Although linkage of mothers to HIV care was significantly higher in urban settings, there was no significant difference in linkage of HIV-exposed babies to EID programs. In general, health workers felt that mothers' perspectives about the benefits of PMTCT motivated them to complete the PMTCT cascade and get linked to chronic HIV care. Motivators and hindrances to enrollment of mothers and babies into care were variable within urban and rural settings. Within urban health facilities, health workers reported that limited male involvement in antenatal HIV testing hindered enrolment into chronic HIV care after PMTCT. In addition, availability of infant feeding and nutrition services in the EID clinic could motivate HIV-infected mothers to return their babies for EID, whereas in the rural setting integration of EID and routine immunization services was highlighted as a potential motivator to utilize the services. Within rural facilities high parity was associated with linkage of babies to EID, while in urban facilities demographic and clinical characteristics were similar among the babies linked and not linked to EID programs. At rural health facilities, health workers felt that stigma and fear of disclosure hindered utilization of chronic HIV/AIDS care services after PMTCT. This was different from a qualitative assessment of antenatal mothers in Kenya, where rural HIV-infected mothers found it easier to disclose HIV sero-status to family members [20]. Whereas the situation might be different in Uganda, it is important to note that our study assessed the health worker perspectives that could differ from mothers' perspective. A previous assessment of pregnant mothers in Uganda showed that mothers who thought their husbands would approve HIV testing, were six times more likely to accept an HIV test compared to those who thought their husbands would not approve [10], [12], [21]. Therefore evaluation of pregnant mothers' perspectives of the determinants for disclosure of HIV status and enrollment into long-term HIV/AIDS care after PMTCT remains relevant to development of robust PMTCT programs. Reports from Uganda previously suggested that uptake of PMTCT services was influenced by limited ability of women to make decisions without approval from their male partners [12]. In a prospective cohort of mothers attending ANC between 1999 and 2009 in Nairobi Kenya, male partner involvement in HIV testing during ANC was associated with a reduced risk of vertical transmission of HIV [12], [22]. Therefore continued efforts to increase involvement of men in antenatal PMTCT services, including HIV testing and EID, could improve uptake of PMTCT interventions during ANC and subsequent enrolment into chronic HIV treatment programs. Linkage of mother-baby pairs to HIV care programs doubled the 8% figure reported in the 2011 national PMTCT program review in Uganda [23]. Higher linkage rates in urban settings was attributed to availability of point of care testing for CD4 and peer mothers that escort clients to chronic care points at urban antenatal clinics, as reported in the qualitative assessment. Our data emphasizes previous reports that availability of CD4 counts, antiretroviral therapy and use of peer mothers increase uptake of PMTCT services [14], [21], [24].

Our data reflects previous reports that mothers in urban and rural settings could be motivated by variable factors, determined by the local norms and practices within the respective regions [21]. Our results emphasize the fact that scale up of PMTCT services and chronic HIV care within health care facilities, requires further evaluation and careful consideration of variable factors that may differ in urban and rural settings. Therefore, national PMTCT programs need adaptation to local socio-cultural contexts that might influence uptake and continued utilization of proven interventions to eliminate new HIV infections among HIV-exposed infants [9], [21]. Similarly, education of mothers about benefits of PMTCT and EID services remains an essential component of the ANC counselling package. Favourable maternal and infant outcomes of maternal ART in the option B plus strategy will likely increase the utilisation of EID and improve linkage of HIV-infected women to HIV care.

Strengths of this study were the inclusion of rural and urban facilities as well as collection of qualitative data to provide additional evidence on factors that may affect linkage to care. Lack of mothers' unique identifiers through the HIV care services within each facility was a limitation; however, our study used mothers' names to track mothers for linkage to the HIV clinics. Babies in the EID registers were identified by their mothers' names. In cases where mothers' names were similar, at least two additional demographic identifiers were used to confirm linkage to HIV care. We were unable to determine whether mothers and their babies were dead or linked to HIV care services at a different health care facility due to lack of a unique identifier for tracking inter-facility patient transfers. We recommend innovations of unique patient identifiers such as national identification numbers that can be used to track mothers and their babies through HIV care programs within the entire region or country. We did not collect data on CD4 count levels as predictors for linkage to care, however, this should not affect the generalizability of our results since ART is recommended for all pregnant mothers irrespective of CD4 counts. In addition, individual mothers' adherence to the antiretroviral drugs offered for PMTCT was not evaluated. In Kwazulu-Natal, 61% (57/94) of antenatal women had good adherence with their PMTCT prophylaxis with triple therapy during pregnancy, although this study does not mention the proportion that remains on therapy after delivery [25]. Therefore, monitoring and evaluation of option B plus PMTCT interventions should consider patient adherence to the drugs provided, in addition to adherence to the program services provided.

## Conclusion

Post-natal linkage of HIV-infected mothers to chronic HIV care and HIV-exposed infants to EID programs is low within health care facilities in Uganda. A third of HIV-infected mothers, a quarter of HIV-exposed babies and an eighth of mother-baby pairs were linked to chronic HIV care and early infant diagnosis care at facilities with comprehensive prevention, care and treatment services. Barriers to linkage to HIV care vary in urban and rural settings. We recommend targeted interventions to rapidly improve linkage in the era of ART for elimination of MTCT.
